# Case Report: Osteosarcoma following traumatic injury in a juvenile Argentine black and white tegu (*Salvator merianae*)

**DOI:** 10.3389/fvets.2026.1820849

**Published:** 2026-05-11

**Authors:** Sungryong Kim, Yerim Kim, Younghoon Jo, Ki-Jeong Na, Dongwoo Chang, Namsoon Lee, Dong-Hyuk Jeong

**Affiliations:** 1 Laboratory of Veterinary Laboratory Medicine, College of Veterinary Medicine, Chungbuk National University, Cheongju-si, Republic of Korea; 2 Section of Veterinary Medical Imaging, College of Veterinary Medicine, Chungbuk National University, Cheongju-si, Republic of Korea; 3 Laboratory of Wildlife and Conservation Medicine, College of Veterinary Medicine, Chungbuk National University, Cheongju-si, Republic of Korea; 4 Wildlife Center of Chungbuk, Cheongju-si, Republic of Korea

**Keywords:** Argentine black and white tegu, computed tomography, cytology, diagnostic imaging, histopathology, osteosarcoma, reptile

## Abstract

Osteosarcoma (OSA) is a malignant neoplasm of mesenchymal origin characterized by osteoid or immature bone production and is rarely reported in reptiles. A 3-month-old Argentine black and white tegu presented with a subcutaneous mass on the left flank, approximately 2 months after a dog bite. Physical and radiographic examination revealed no initial internal abnormalities, but follow-up examination revealed a rapidly enlarging soft tissue mass. Ultrasonography confirmed a well-encapsulated mass, while contrast-enhanced computed tomography demonstrated mineralization, rib osteolysis, and multiple bilateral nodules, indicating a malignant process. Histopathological examination of the excised mass demonstrated features consistent with giant cell-rich osteosarcoma, including irregular bony trabeculae, numerous multinucleated giant cells, and peripheral osteoid matrix. This case highlights the diagnostic challenges of OSA in reptiles, the value of integrating diagnostic imaging and histopathology, and the need for species-specific imaging protocols in ectothermic species, thereby expanding the literature on reptilian neoplasia.

## 1 Introduction

The Argentine black and white tegu (*Salvator merianae*) is a South American reptile inhabiting regions south of the Amazon, that has traditionally been used for meat and leather and is now recognized as one of the increasingly popular reptile species in the global exotic pet trade (1, 2).

Sarcomas are a diverse group of malignant tumors arising from mesenchymal tissues, including bone, cartilage, fat, muscle, and vascular tissues (3). Among these, osteosarcoma (OSA) is the most common primary bone malignancy, characterized by the production of osteoid tissue or immature bone by malignant cells (4).

In reptiles, neoplastic diseases have become increasingly recognized due to improved veterinary care and longer lifespans in captivity (5). However, reports of OSA in reptiles remain rare, with documented cases in snakes, lizards, and turtles, often identified via radiographic imaging followed by histopathology (6, 7). These tumors may present as localized swelling, lameness, or pathological fractures. Despite their rarity, OSAs in reptiles share some histological features with those in mammals, although species-specific differences in tumor behavior and progression remain poorly understood. A review of reptilian neoplasia cases found that sarcomas comprised 6.68% of all tumors, and OSAs only 2.3%, with reports in one central bearded dragon (*Pogona vitticeps*), two green iguanas (*Iguana iguana*), and two spiny-tailed monitors (*Varanus acanthurus*) (7).

Due to the limited available literature on reptilian OSA, insights from canine and feline OSA are often referenced for clinical context. In dogs, OSA is characterized by aggressive biological behavior and a high metastatic rate, whereas feline OSA generally exhibits less aggressive behavior and a more favorable prognosis (8–10).

This case report presents OSA in a black and white tegu, with a focus on cytology, histopathology, and computed tomography findings. Given the rarity of OSA in reptiles, this case may contribute to a better understanding of the disease, enhancing diagnostic approaches for this condition.

## 2 Case description

**Figure 1:**
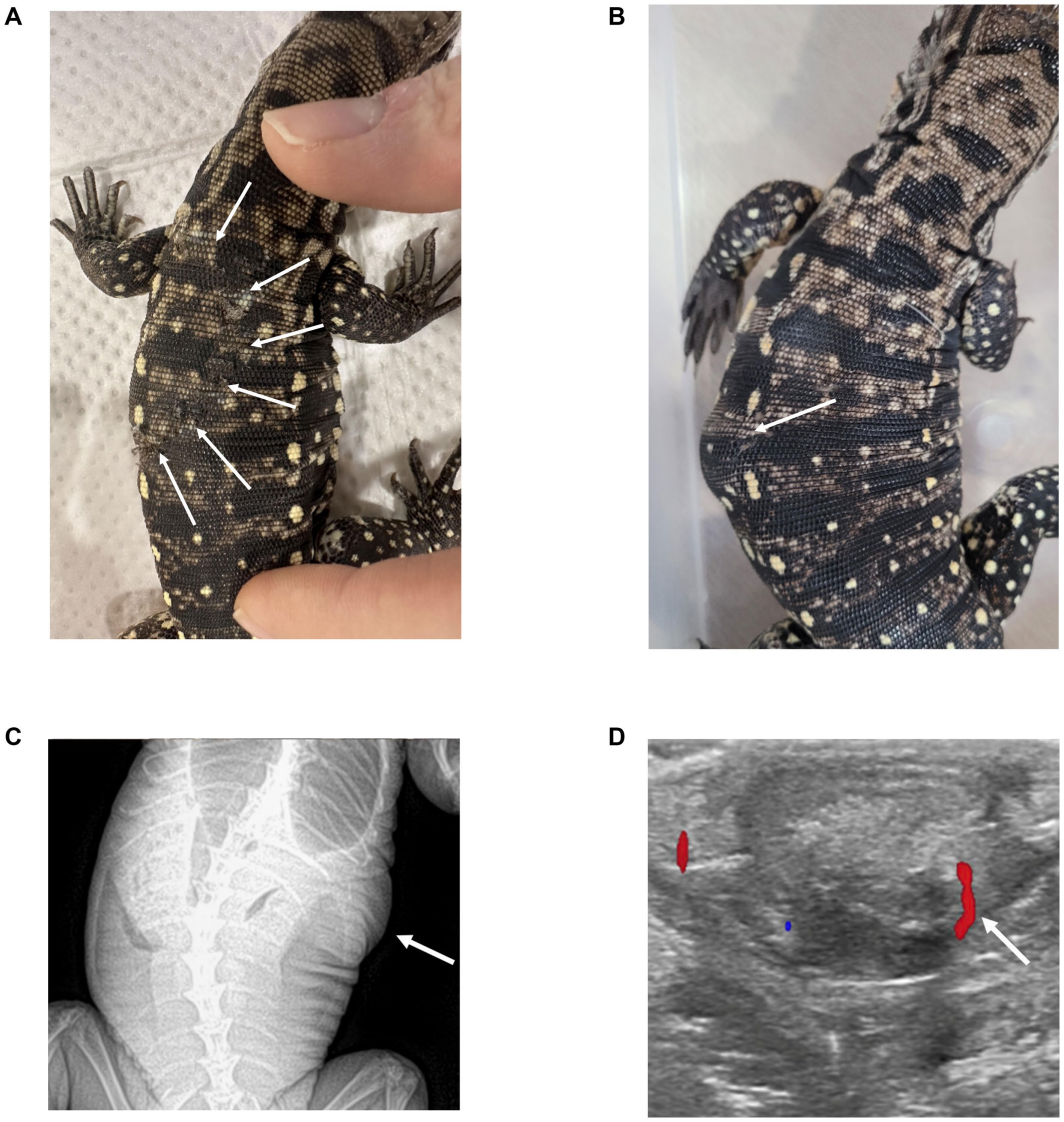
Sequential photographic and imaging findings in an Argentine black and white tegu with a subcutaneous flank mass. **(A)** Initial dog bite with visible scale wounds (arrows). **(B)** At 77 days, a rapidly enlarging mass developed at the original bite site (arrow). **(C)** Follow-up DV radiograph obtained 77 days after presentation, showing a dome-shaped soft tissue opacity in the left flank region, continuous with the cutaneous layer (white arrow). **(D)** Color Doppler ultrasonography demonstrates a well-defined, encapsulated mass with mild intralesional vascular flow (white arrow).

A 3-month-old Argentine black and white tegu (*Salvator merianae*) was presented to the veterinary teaching hospital after a dog bite. Physical examination and radiographic evaluation revealed superficial scale injuries without evidence of musculoskeletal or internal abnormalities (Figure 1A). Based on these findings, the patient was discharged without specific treatment and monitored for progression. Seventy-seven days later, the patient was re-presented with a rapidly enlarging mass at the site of the previous bite wound (Figure 1B).

Dorsoventral view radiographs (Figure 1C) showed a dome-shaped soft tissue mass associated with the cutaneous tissue of the left flank. Ultrasonography identified a single, well-defined, well-encapsulated mass measuring 17.2 mm × 13.6 mm with mixed echogenicity and mild intralesional vascularity (Figure 1D). No evidence of herniation or communication with abdominal viscera was observed.

**Figure 2:**
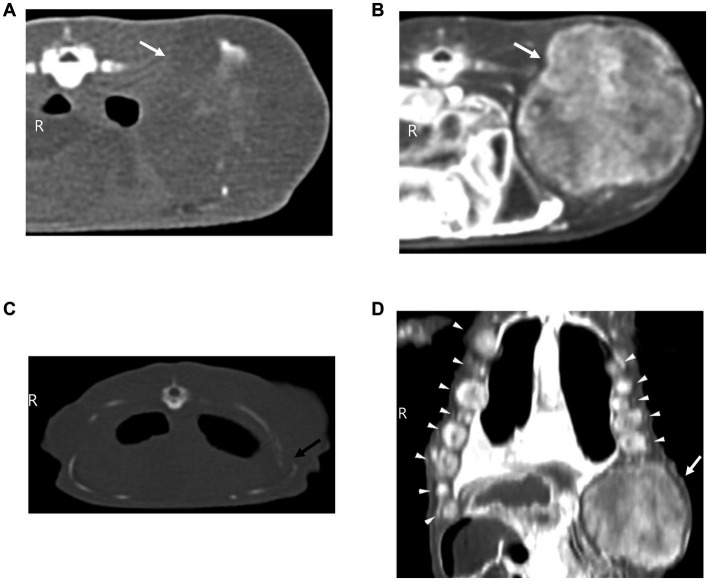
Computed tomography (CT) evaluation of the flank masses. **(A,B,D)** Soft tissue images and, **(C)** Bone window image. **(A)** Transverse pre-contrast CT image of the dominant left flank mass (white arrow), showing internal mineralization and hypoattenuating regions. **(B)** Corresponding post-contrast transverse image showing strong, heterogeneous contrast enhancement of the same mass (white arrow). **(C)** Transverse CT image showing osteolysis of the 10th rib (black arrow). **(D)** Post-contrast dorsal CT image showing multiple enhancing nodules along the bilateral costal margins (arrow heads) and left flank mass (white arrow).

Contrast-enhanced computed tomography (CT) was performed under general anesthesia using a 16-slice CT scanner (Aquillion 16, Toshiba Medical Systems Corporation, Otawara, Japan) at 150 kVp and 120 mA, with a slice thickness of 1 mm for both pre- and post-contrast phases. The patient was induced with 5% isoflurane and catheterized via a 24 G catheter in the ventral coccygeal vein. Iohexol (Omnipaque, GE Healthcare) was administered intravenously at a dose of 3 mL/kg (based on a body weight of 410 g), and post-contrast imaging was performed 4–5 min post-injection. CT revealed a large, well-defined left flank mass (2.88 cm × 2.86 cm × 2.56 cm, W × L × H) with mixed internal calcification (Figure 2A), heterogeneous contrast enhancement (Figure 2B), and adjacent rib osteolysis (Figure 2C). Marked post-contrast enhancement in both hypodense and hyperdense regions (pre-contrast: 12–164 HU; post-contrast: 452–566 HU) indicated high vascularity and active tumor perfusion, consistent with a malignant process. Additionally, multiple enhancing nodules were observed bilaterally along the body wall, ranging from 3.8 to 8.8 mm and exhibiting contrast patterns similar to the dominant mass (Figure 2D). Their rapid growth and associated osteolysis supported a malignant etiology. No pulmonary lesions were identified. Nevertheless, considering the patient’s history of a recent bite wound and young age, an inflammatory or reactive lesion secondary to trauma could not be entirely excluded. Therefore, a conservative approach was initially adopted, and prednisolone (2 mg/kg), ursodeoxycholic acid (10 mg/kg), and famotidine (0.5 mg/kg) were administered orally once daily for 1 week, with close follow-up planned. However, due to lack of response, the prednisolone was increased to 3 mg/kg and continued for an additional 1 week. Despite this, the mass continued to grow rapidly and new flank lesions appeared. To alleviate discomfort and enable thorough histopathological evaluation, surgical excision of the left flank mass was performed.

Anesthesia was induced with 5% isoflurane and maintained at 2%–5%. Saline (0.9%) was infused via a coccygeal catheter at 30 mL/kg/day. The patient was intubated with a 6 Fr tube and placed in ventral recumbency. After blunt dissection, the mass was resected using monopolar electrosurgery. Complete excision was not possible due to adhesions. The wound was closed with 4–0 nylon mattress sutures. The specimen was submitted for histopathology.

**Figure 3:**
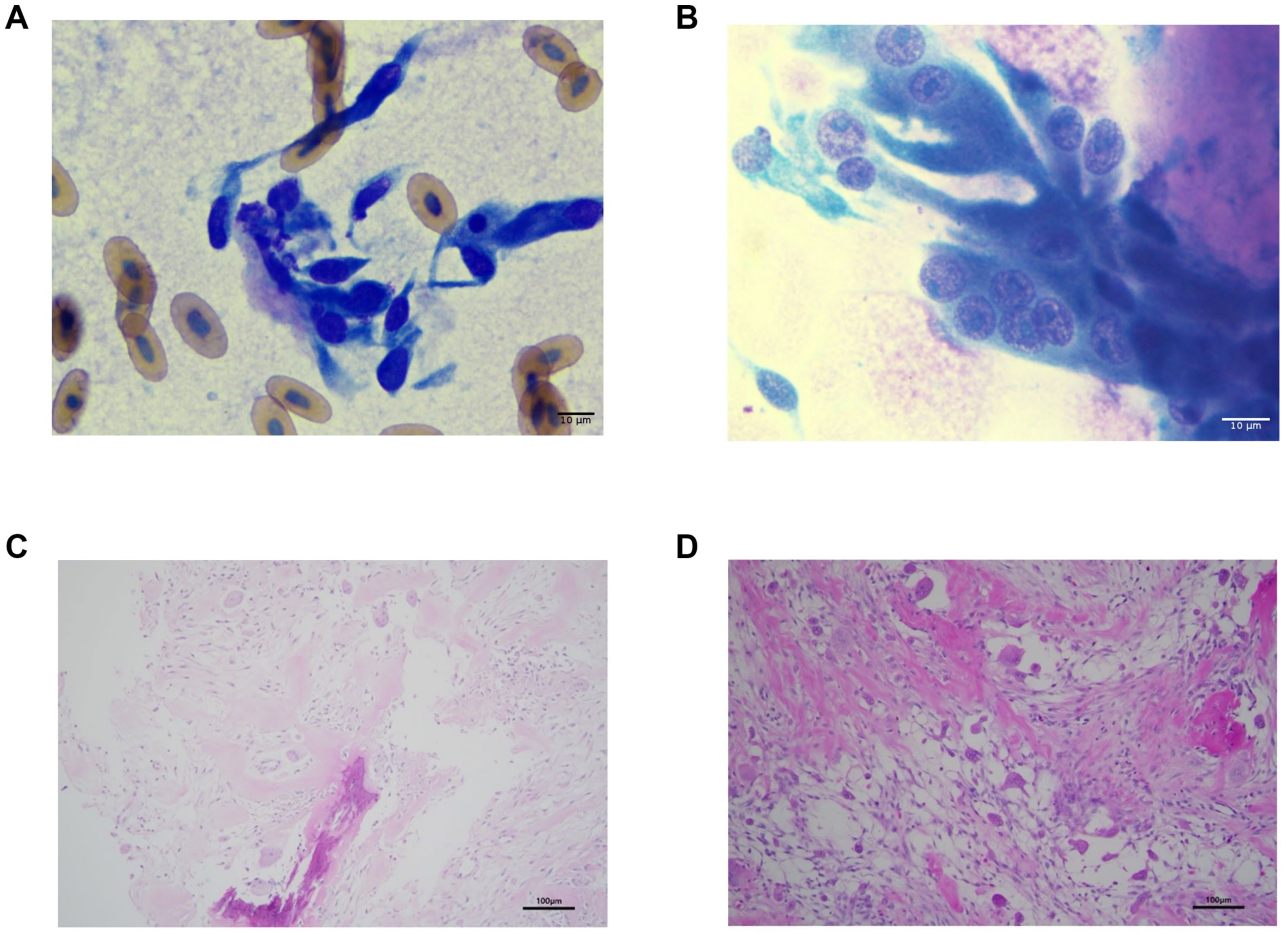
Cytologic and histopathologic findings of the subcutaneous mass. **(A)** FNA smear showing elongated mesenchymal cells without malignant features. **(B)** Tru-Cut biopsy smear revealing multinucleated spindle cells with nucleoli and surrounding eosinophilic osteoid. **(C)** Histopathological findings from the Tru-Cut biopsy showing multinucleated giant cells presumed to be osteoclasts and eosinophilic, homogeneous extracellular material identified as osteoid were observed. **(D)**. Histopathology of the surgically excised mass demonstrating central ossification and peripheral osteoid with multinucleated cells in empty spaces. **(A,B)**, Modified Wright-Giemsa stain, ×1,000, **(C,D)**, H&E stain, ×200.

Cytology of the mass revealed elongated spindle cells without atypia, suggesting fibrosis or granuloma related to bite wound healing (Figure 3A). In contrast, a Tru-Cut biopsy smear showed multinucleated spindle-shaped mesenchymal cells with prominent nucleoli, surrounded by eosinophilic homogeneous extracellular material (osteoid), suggesting a malignant neoplasm of osseous origin (Figure 3B).

Histopathology of the Tru-Cut biopsy showed infiltrative spindle cells in short streams within a thin collagenous stroma, exhibiting anisocytosis, anisokaryosis, and multinucleation. The cells had indistinct borders, pale eosinophilic cytoplasm, and ovoid nuclei with stippled chromatin and prominent nucleoli. Neoplastic cells were entrapped within eosinophilic osteoid matrix (Figure 3C), but some were suspected osteoclasts, complicating definitive diagnosis.

Histopathological examination of the excised mass revealed a fibrous capsule surrounding a lumen filled with atypical cells. The central area contained irregular bone trabeculae with loosely arranged spindle cells. The peripheral region showed proliferating spindle to polygonal cells associated with abundant osteoid-like material. Scattered multinucleated giant cells were observed, and mitoses were rare, occurring at 1–2 per 10 high-power fields (Figure 3D).

## 3 Discussion

This case describes a rare occurrence of OSA in a juvenile Argentine black and white tegu, arising at the site of a previous bite wound. Diagnosis was based on cytological, histopathological, and diagnostic imaging findings. Although neoplasia is rare in reptiles, the clearly documented trauma preceding tumor formation highlights a potential association between injury and malignant transformation. Additionally, the emergence of contralateral flank masses suggests possible systemic involvement or multifocal progression.

The relationship between trauma and malignant tumor formation remains controversial. However, clinical and experimental evidence indicates that significant tissue injury, chronic inflammation, and foreign body reactions may contribute to neoplastic transformation (11–13). In human medicine, several sarcomas have been reported at sites of previous trauma (14, 15). Experimental studies have further demonstrated that direct skeletal muscle injury can promote soft tissue sarcoma formation in genetically predisposed murine models, particularly in the presence of p53 deletion or oncogenic KRAS activation, supporting an initiator–promoter model of sarcogenesis in which trauma enhances tumor development at the site of injury (16–18). Nevertheless, the multiple nodules observed on the contralateral right flank could not be definitively characterized due to the lack of histopathological confirmation, which represents a limitation of this study. These lesions were located distant from the original injury site and showed a relatively symmetrical distribution. This pattern makes a direct trauma-induced origin unlikely and instead suggests possible systemic involvement or multifocal progression.

Several mechanisms may account for these findings. OSA is a highly metastatic tumor in veterinary patients, particularly in dogs. Most cases are considered to harbor micrometastatic disease at the time of diagnosis, and metastasis most commonly occurs in the lungs, followed by other bones and visceral organs (19–21). Information regarding the biological behavior and metastatic patterns of osteosarcoma in reptiles remains extremely limited. Direct extrapolation should therefore be made with caution. Metastatic spread from the primary tumor is considered the most likely explanation in this case. Multicentric tumor development or systemic processes related to osteoblastic or osteoclastic activity cannot be excluded. Further investigation, including histopathological evaluation of these nodules, is required for definitive characterization. Cytological evaluation initially revealed only spindle-shaped mesenchymal cells without overt malignant features. Tru-Cut biopsy smears demonstrated multinucleated giant cells resembling osteoclasts and eosinophilic osteoid-like material, although malignant osteoblasts were not clearly identified. Osteosarcomas typically exhibit moderate to high cellularity and may contain malignant osteoblasts appearing individually or in clusters, often mimicking reactive osteoblasts but showing anisocytosis, anisokaryosis, prominent nucleoli, and basophilic cytoplasm (22). Multinucleated giant cells are also commonly observed in lytic bone lesions and may complicate cytologic interpretation. It is also worth noting that prednisolone was administered prior to definitive diagnostic sampling. Prednisolone was initially given at 2 mg/kg once daily for 1 week, then increased to 3 mg/kg for an additional week due to lack of response, resulting in a total treatment period of 2 weeks before surgical excision. In human medicine, glucocorticoids have been shown to exert cytotoxic effects on certain cell populations and alter the histological composition of lesions, potentially compromising diagnostic accuracy in lymphoid neoplasms (23, 24). Although direct evidence in reptilian bone tumors is lacking, a similar effect on cytological findings cannot be entirely excluded in the present case. However, given the inherent cytological characteristics of GCRO, in which osteoclast-like giant cells predominate and malignant osteoblasts may be inconspicuous, it remains uncertain whether the bland cytological appearance on initial fine-needle aspiration was attributable to prior glucocorticoid administration or to the nature of the tumor itself.

Histologically, the excised mass contained numerous multinucleated giant cells. Osteosarcomas are classified into subtypes including osteoblastic, chondroblastic, and fibroblastic forms, as well as rarer variants such as giant cell rich osteosarcoma (GCRO), which accounts for approximately 1%–3% of cases in mammals (25). GCRO is characterized by abundant osteoclast-like giant cells and relatively scant tumor osteoid (26). To the author’s knowledge, however, GCRO has not been previously reported in reptiles.

Advanced imaging, including ultrasonography and CT, provided critical information for evaluating the lesion. Ultrasonography confirmed the subcutaneous location and excluded herniation but lacked sufficient resolution to fully characterize the lesion. Contrast-enhanced CT revealed a well-defined mass with heterogeneous enhancement, mineralization, rib osteolysis, and multiple enhancing nodules along the body wall. These findings strongly suggested malignancy.

Although only pre- and post-contrast phases were acquired in this case, the image likely captured a state of over-enhancement, which complicated interpretation. Two physiologic factors may explain this outcome. First, reptiles exhibit relatively slow metabolic and circulatory dynamics, which delay vascular perfusion and interstitial contrast clearance (27). Second, reptiles possess a renal portal system, wherein substances administered via the ventral coccygeal vein may first pass through the kidneys before entering systemic circulation (28). This additional delay in systemic distribution can further increase the likelihood of excessive pooling of contrast medium at the time of imaging. Combined, these features may obscure the distinction between necrotic, cystic, and viable tissue components, highlighting a limitation of CT protocols to reptiles. Therefore, further research is needed on customized CT protocols based on physiological circulation studies of reptiles.

In conclusion, this case illustrates a rare occurrence of osteosarcoma arising at a previous traumatic injury site, suggesting a potential association between significant tissue damage and malignant transformation in reptiles. This case also highlights the diagnostic challenges of OSA in reptiles, the value of integrating diagnostic imaging and histopathology, and the need for species-specific imaging protocols in ectothermic species and expands the literature on reptilian neoplasia.

## Data Availability

The original contributions presented in the study are included in the article/supplementary material, further inquiries can be directed to the corresponding authors.
